# Ulceration of the Os Uteri

**Published:** 1858-02

**Authors:** J. N. Graham


ARTICLE II.

### ULCERATION OF THE OS UTERI.

READ BEFORE THE COOK COUNTY MEDICAL SOCIETY, AT CHICAGO.

BY J. N. GRAHAM, M. D.

On a question like the one before us, upon which there is the widest diversity of opinion—a difference, too, not between the true physician and the mere pretender, or the man of science and experience, but between minds of the highest grade of intellect, and the best advantages for testing, each the *truth* of the position he assumes, it is no easy matter to come at the truth; and we are far from arrogating to ourselves the right to decide in which of the varied opinions now so earnestly contended for lies the real merit or the unqualifiedly correct method of treating the diseases in question. Nevertheless, it is the prerogative of the unbiased learner to view this or any question of dispute in all its bearings, with all its *pros* and *cons.* The truth of any system is best developed and brought to light by thorough investigation. And yet, on many questions, it is difficult to determine which is right, and why men of enlarged experience should so widely differ. But, alas! for the best of men, it is human to err. The highest of intellects are not free from bias; and men often, in the science of medicine, as in other things, set up for themselves an imaginary image, and then bend every energy to clothe it in form, and give it comely proportions. Every theory or ism that has had existence, from the crudest notions first formed in the far off ages, when our science had but its faintest beginnings, to the present, has had its advocates. Each supposed that *this* alone was right, and *that* was wrong. As in the disease before us, one party assumes that as the *cause*, which the other regards only as the *effect;* and thus each argues from his assumed basis, and *on* that basis (which may be wrong or not) goes on to prove, by statistics and argument, the validity of his position.

Bennet will tell you that "inflammation and ulceration of the neck of the uterus are, in the majority of cases, the real cause of morbid uterine changes and symptoms, and that a little reflection will show that this must be the case." And to prove this, he gives you in array three hundred well marked cases treated by him in the Western Dispensary, in London, every case minutely examined and reported, with the results of treatment.

On the other hand, Dr. Ashwell, occupying similar advantages in Guy's Hospital, will tell you that not more than twenty cases of ulceration of the cervix uteri in a thousand occurred. Again, West will summon before you a long catalogue of cases possessing all the diagnostic signs of ulceration of the os uteri, and prove to you most conclusively that in between forty and fifty per cent of these cases, marriage, pregnancy, abortion or delivery, are referred to as the *primary* cause, and that, if ulceration exists at all, it is but the consequent upon these or some other cause, and not itself the producing cause of all the other symptoms.

In this state of things, it would appear at least that the data from which we have to draw our conclusions is in a very imperfect state, and that, for the *truth* of the question, we have to form our opinion from observation, and from what we can gain from the conflicting elements before us. But a superficial glance at the anatomical and physiological structure of the uterus will give us some clue to its amenability to disease. Hanging pendulus, as it is, suspended almost entirely by ligaments in the pelvic cavity, its venous structure devoid of valves, thus impeding the return of blood from its extremity, and increasing the tendency to congestion; while its *body* is composed of dense muscular fibre, the cavity of which is lacking in cellular tissue, lined by a nearly elementary mucous membrane, thus making it less susceptible of inflammation. Its *neck*, on the contrary, contains cellular tissue, is of a less dense structure, more vascular, and has its cavity lined by a thick vascular membrane, "studded by numerous mucous follicles," every way susceptible to inflammation and ulceration.

When we remember, also, that the uterus receives a large portion of its nerves from the spermatic, aortic and hypogastric plexuses, thus being left almost entirely to the control of the sympathetic nerves, and subject as it is to the various changes consequent upon the important functions it has to perform in the economy of nature, we shall not wonder that it is more or less amenable to the disease under consideration. Still, from all this, we are no better prepared to account for the variety of opinion in regard to the extent or importance of the specific ailment before us. We can only answer as before, that, having once established or assumed a position, the tendency is to make everything else subservient to that. Hence, that alone is regarded as truth which seems to substantiate and fortify the position taken. It is a glorious excitement for the adventurer to strike out upon some new and untried path, and leave others but to humbly tread in the highway to eminence he has marked out. But, alas for the aspirant! his fancied visions often fly before the light of truth, like the shadows before the coming morning. That there is such a disease as ulceration of the os uteri, no practitioner will deny; and it is equally as clear to our mind, that in many this is the *cause* of the general ailment for which we are often called to prescribe, and that *local* treatment will remove the cause, and the patient recover. There can be no impropriety in assuming that the various sources of irritation consequent upon the female existence, such as catamenia in all its varied and irritating forms, marriage, abortion, delivery, etc., are in themselves fruitful sources of inflammation and ulceration of the os uteri, and that from some one or all of these causes we may find it to exist.

But the skillful physician will discriminate between symptoms and results that arise from merely local causes, and those that are the result of *general* ailment, and will adopt his treatment accordingly.

Within the last few years there seems to have existed a *womb-burning* mania; and no sooner does the female of pallid countenance and hypochondriac tendency complain of "weakness in the back, depression of spirits, irregular or painful menses, leucorrhœal discharges, bearing down pains," and so on, than the cause is at once divined, and, forsooth, she must be burned! A new discovery has been made in the healing art, and nothing will answer but she must submit to a long series of painful treatment. Perhaps she recovers—perhaps not; but if not, "the disease was incurable; she had gone too long" before the distinguished pretender was called; and leaving her with this poor consolation, duped by her simplicity and his duplicity and ignorance, he hurries on to another, and yet another victim. His popularity and means of gain but increased by the number he victimizes. Alas! that such should be true of any who holds a position in the medical profession. But, we blush to say it, the fact, in some cases, is undeniable.

We have in our mind a practitioner residing in another city, claiming the position of a respectable physician, and holding an enviable position in point of practice, who, for the five or six years with which we were acquainted with him, was never without a large number of such cases. Indeed, he usually reported more such under treatment than all the other physicians together, in a population of some twenty thousand. At one time he was known to have twenty-one such cases under treatment at once, all on one ill-fated street. And this is but a specimen of the way in which many, of late, have ridden into popularity.

Alas! for fallen humanity—for our profession! It only proves that the longer the ears, the more obtuse and brazen-fronted the brain, the nearer approximation to the animal called *ass*, the surer his prospects of success.

We said the judicious physician will discriminate between the cases brought before him, and adopt his treatment accordingly. Ascertaining the *cause*, he will go to work to remove it, wisely selecting the means to the end. He will judge between the symptoms that arise from local irritation, where the general health is not impaired, and those that are the result of general derangement. In the one case, a few well-directed astringents or escharotics applied, or, it may be, emollient washes, and strict adherence to cleanliness on the part of the female herself, will produce a cure when constitutional treatment would appear superfluous. In the other case, his patient reduced in strength, anæmic, with all the symptoms of a vitiated and deteriorated state of the system, with marked ulceration of the cervix uteri, requires *general* as well as *local* treatment. Here he goes to work to restore the constitution to a healthy condition. The digestric functions are regulated, and the general system is built up by tonics and chalybeates, such as quinine, syrup, iodide of iron, iron combined in any of its forms, regular habits, fresh air, generous diet, etc., with local applications when necessary—in short, a treatment based on general principles, until jaded nature is restored to its normal condition. We have on hand some such cases—one of malignant ulceration, with general ill-health. The patient informed us she had been ill of uterine difficulties, and had been treated for such for eight years of her married life, had often conceived, but as often miscarried; indeed, she looked like a living bed-ridden specimen of disease and hypochondriacism. She had never borne a living child. Under constitutional and local treatment for about three months, she recovered, and has since given birth to two living, healthy children. We can also bear evidence to some such cases, as reported by Bennett, of ulceration treated successfully, during the first months of pregnancy, by applications to the diseased parts, and of other cases, where little else was done than to pursue a constitutional treatment. And why not ulceration of the cervix uteri be cured by constitutional treatment, as well as aptha, or the ulcers on the cheeks and tongue in stomatitis materna. Do we not often find the same deteriorated state of the system, brought about by the same causes, or induced by a scrofulitic tendency, in the one case that we do in the other? In stomatitis, for instance, we have general derangement of the economy—a breaking down of the fibrous structure of the blood—a want of albumen, and all the healthy constituents in its vitalization. Where we find defibrination of the blood, with the collateral constitutional ailments that generally follow, may we not with an almost certainty look for a diseased mucous surface? In the "nursing sore mouth" of the mother, with wan cheek, emaciated form, deranged appetite, indigestion, nervous sensibility and general debility, no physician posted in his profession would think of curing his patient by applications or washes for the mouth, while the general system was neglected. By treating the patient constitutionally, the ulcers of the mouth would heal. The same will follow in multitudes of cases where there is ulceration of the uterus, keeping the parts clean by soothing emulsients or pure water.

The ten thousand injurious habits of society now-a-days—such as dress, diet, irregular hours, over-heated and badly-ventilated rooms, with the vices common in the lower grades of society—are all calculated to undermine the female constitution, and increase the frequency of this disease; and the physician who relies on local treatment alone will come sadly short of relieving the malady.

We find, as we have said, a great discrepancy in the hospital reports—a difference in the *extent* of the disease and the treatment best adapted to its cure. Statistics have been piled up before us to fortify whatever position was assumed or deemed to be right, without regard to the source from whence the material was taken. In the hospitals of London and Paris, for instance, are collected the common prostitute, the low and the vile, from the very dregs of society. In these we might expect this disease, local and constitutional; and in large numbers reported, the treatment was both local and constitutional: so that it may be a question how much to attribute to one method or the other, or to both united. As reported by Ashwell, most of the cases, with all the constitutional symptoms of ulceration recovered under general treatment. We believe the data from whence our hospital reports are taken are not to be relied on as furnishing a correct estimate of the extent of the disease, or the comparative number of females afflicted, from the fact that the material there collected is such that would engender this disease from the very causes existing in itself. And again, each reporter, anxious to establish his own position, may easily find material for doing so.

The question of the magnitude and extent, or importance of this disease, may yet be a subject for further investigation. And still, we see no good reason why there should be such a diversity of opinion on a matter so amenable to the practice indicated by common sense. We enter our protest against any system that would make it a hobby upon which to ride into popularity; and we are very suspicious of the skill or honesty of any physician who from day to day multiplies his number of cases, and exults in his success in cauterization. Let us be governed, in our treatment of this and all other diseases, by great general principles. Then we will have no hobbies to ride; our treatment will be governed by the exigencies of the case, and the results, based upon the true foundation of science, will bear investigation.

